# Late-life depression: Epidemiology, phenotype, pathogenesis and treatment before and during the COVID-19 pandemic

**DOI:** 10.3389/fpsyt.2023.1017203

**Published:** 2023-04-06

**Authors:** Yuanzhi Zhao, Xiangping Wu, Min Tang, Lingli Shi, Shuang Gong, Xi Mei, Zheng Zhao, Jiayue He, Ling Huang, Wei Cui

**Affiliations:** ^1^Ningbo Kangning Hospital, Ningbo, Zhejiang, China; ^2^Department of Neurology, Ningbo Rehabilitation Hospital, Ningbo, Zhejiang, China; ^3^Ningbo Key Laboratory of Behavioral Neuroscience, Zhejiang Provincial Key Laboratory of Pathophysiology, Translational Medicine Center of Pain, Emotion and Cognition, School of Medicine, Ningbo University, Ningbo, Zhejiang, China

**Keywords:** late-life depression (LLD), epidemiology, phenotype, pathogenesis, treatment, COVID-19 pandemic

## Abstract

Late-life depression (LLD) is one of the most common mental disorders among the older adults. Population aging, social stress, and the COVID-19 pandemic have significantly affected the emotional health of older adults, resulting in a worldwide prevalence of LLD. The clinical phenotypes between LLD and adult depression differ in terms of symptoms, comorbid physical diseases, and coexisting cognitive impairments. Many pathological factors such as the imbalance of neurotransmitters, a decrease in neurotrophic factors, an increase in β-amyloid production, dysregulation of the hypothalamic-pituitary-adrenal axis, and changes in the gut microbiota, are allegedly associated with the onset of LLD. However, the exact pathogenic mechanism underlying LLD remains unclear. Traditional selective serotonin reuptake inhibitor therapy results in poor responsiveness and side effects during LLD treatment. Neuromodulation therapies and complementary and integrative therapies have been proven safe and effective for the treatment of LLD. Importantly, during the COVID-19 pandemic, modern digital health intervention technologies, including socially assistive robots and app-based interventions, have proven to be advantageous in providing personal services to patients with LLD.

## 1. Introduction

In 2019, more than 9% of the world’s population was over 65 years old, and this proportion is expected to increase to 16% by 2050 (1). The physical and mental health of older adults is important because of the various environmental and social stressors in the modern world. The COVID-19 pandemic has significantly impaired the mental health of older adults (2). Although driven by aging, physical disease, and psychological characteristics, late-life depression (LLD) differs from adult depression in terms of its epidemiology, phenotype, pathogenesis, and clinical treatment (3).

## 2. The epidemiology of LLD

The prevalence of LLD significantly varies worldwide (4). Recent epidemiological meta-findings involving 57,486 older adults showed that the average expected prevalence of LLD is 31.8%. In the subgroup analysis, the pooled prevalence was higher in developing countries (40.78%) than in developed countries (17.05%) (5). The prevalence of LLD ranged from 6.3 to 46.6% in Canada, Brazil, Colombia, and Albania (6). Older adults with depressive symptoms accounted for 19.47% in Western countries (7). The prevalence of depressive symptoms in community-dwelling healthy older populations of Australia and US was 9.8% (8), and in mainland China it ranged from 1.5 to 7.9% (9, 10). The prevalence of LLD might be differ among populations. Latinxs had higher odds of LLD than non-Latinx Whites in the US (11). In Canada, the prevalence of LLD was 19.1, 24.2 and 11.9%, respectively, in home and community care, long-term care and palliative home care (12). Moreover, the comorbid of psychological disorders might also affect the prevalence of LLD (13). The prevalence of comorbid anxiety disorders in LLD was 7.4% for generalized anxiety disorder, 4.7% for panic disorder, 5.3% for agoraphobia, 1.1% for social phobia, 2.1% for obsessive-compulsive disorder and 3.7% for post-traumatic stress disorder, with an overall prevalence of 16.84% (14). In addition, LLD were associated with 20−30% of Alzheimer’s type dementia, 20−50% of Parkinson’s type dementia and 50% of vascular dementia, fronto-temporal dementia, and Lewy body dementia (15). And substance abuse disorder largely increases the prevalence of LLD because of its neurotoxic and neuropathological changes on patients (16).

Late-life depression results in an overall economic burden (17). Health service utilization, including direct and indirect healthcare utilization costs, is the main reason for the excessive economic burden caused by LLD. Compared with non-LLD patients, the total medical expenses for LLD patients including outpatient care and hospitalization have increased by one-third (18). In European countries, the total costs of health-related resources for LLD patients were 3,748 euros, while the total cost for non-LLD patients is 3,090 euros (19).

Age and sex are the main risk factors for LLD (20). Advanced age increased the risk of depression in an inverted J-shaped relationship, with an odd ratio (OR) of approximately 75. Compared with older adults below 70 years of age, the middle-old (70–80 years) population had higher odds of depression, while the oldest-old (> 80 years) population was not at an increased risk of depression (21). Moreover, a national survey in the USA demonstrated that significantly more depressive symptoms were observed among women (26.9%) than men (19.9%) (22). Additionally, marital status and living conditions were associated with the risk of LLD. A large-scale cross-sectional study in Japan emphasized the relation of LLD to “separation/divorce” and “debt,” possibly because loneliness after separation/divorce might cause depression, while debt represents a major adverse event that can increase the likelihood of LLD (23, 24). A study of older adults in rural China found an association between high CESD-10 scores (center for epidemiological studies depression scale-10, indicating symptoms of depression) and low annual personal income, polluted cooking fuel, toilets without seats, and a lack of bathing facilities (25). Consistent with this, a study in Japan showed that socioeconomic status may permanently affect the future incidence of LLD, and that retirement increases the occurrence of LLD (26, 27).

The infection of COVID-19 could lead to psychological consequences, including depression, anxiety, stress and adjustment disorders, poor sleep, and increased substance use (28). Depression was among the most commonly reported symptoms in COVID-19 survivors without pre-existing mental health problems (29). A study in England found that the prevalence of clinically significant depressive symptoms among older adults increased from 12.5% (before the COVID-19 pandemic) to 22.6% (June and July 2020), and further increased to 28.5% (November and December 2020) (30). The Irish Longitudinal Study on Ageing (TILDA) examined trends in depressive symptoms among older adults before and during the COVID-19 pandemic; the prevalence of clinically significant depressive symptoms accounted for 19.8% during the COVID-19 pandemic, which was more than two times higher than before the COVID-19 pandemic (31). A recent study compared trajectories of depressive symptoms before and after contracting COVID-19 between matched long- and short-COVID groups. Depressive symptoms increased immediately after the onset of the infection in both groups. But the long-COVID group showed substantially greater initial increases in depressive symptoms and heightened levels over 22 months of follow-up (28). And during the COVID-19, the number of LLD patients largely increased, especially in patients with other chronic diseases. The prevalence of depression increased from 17 to 33% in diabetic patients during the COVID-19 pandemic (32). The main risk factors associated with LLD that emerged during the COVID-19 pandemic were sex, loneliness, poor sleep quality, and poor motor function (33).

**FIGURE 1:**
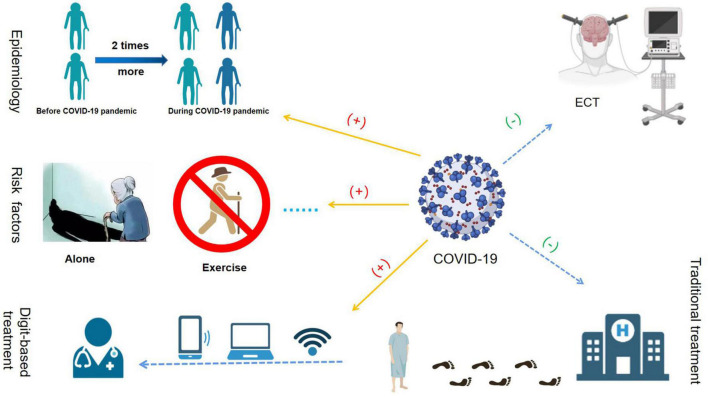
The effects of the COVID-19 pandemic on late-life depression (LLD). The COVID-19 pandemic increased the prevalence of LLD significantly. The prevalence of clinically significant depressive symptoms during the COVID-19 pandemic, which was more than two times higher than before the COVID-19 pandemic. The main risk factors associated with LLD during the COVID-19 pandemic were female sex, loneliness, poor sleep quality, and poor motor function. During the COVID-19 pandemic, owing to the possibility of virus transmission *via* bag-mask ventilation in the electroconvulsive therapy (ECT) procedure, many ECT units in hospitals worldwide were closed. During the COVID-19 pandemic, the conventional medical and healthcare service mode was “devastating,” while modern digital health intervention technology developed rapidly.

Late-life depression is a significant public health problem owing to its high prevalence and complex risk factors. Population aging and the global COVID-19 pandemic have adversely affected the physical and mental health of older adults (Figure 1). Therefore, it is critical to understand the unique phenotype and pathogenesis of LLD and investigate effective and safe treatments for this disease.

## 3. The unique phenotypes of LLD

Although the diagnostic criteria for LLD and adult depression are similar, the clinical presentations between LLD and adult depression tend to vary widely.

Central depressive symptom networks differed between the LLD and adult groups. Loss of interest and depressed mood were central to the depressive symptom network structure in both older and younger adults. Additionally, wishes for death and pessimism were the central depressive symptoms in the network of older adults, while fatigue and appetite changes were consistently found among the depressive symptoms in young adults, but not in older adults (34). Moreover, compared with adult depression, LLD is mainly characterized by lower mood and more physical discomfort symptoms. Loss of appetite and weight, insomnia, and difficulty falling asleep are also common among older adults (35). Other unique features of LLD are indifference, weakened interest in all aspects of life, and guilt (36).

Compared to adults with depression, patients with LLD have a higher rate of concomitant drug use. A systematic review and meta-analysis of 16 observational studies that followed nearly 500,000 people showed that the OR for depression was high among individuals with diabetes (37). Due to their physical illness, LLD patients are also likely to be exposed to multiple drugs, which may increase their symptoms of depression. Regarding patients with cerebrovascular disease, the use of calcium channel blockers and nitrate esters is positively associated with a higher risk of depression (38). In addition, complex polypharmacy easily results in drug interactions and multiple adverse effects (39). Therefore, extreme caution should be exercised when prescribing antidepressants to patients with LLD and heart, liver, or kidney disease.

Coexisting cognitive impairments are common in patients with LLD. Approximately 30% of LLD patients exhibit abnormal performance in verbal fluency, response inhibition, novel problem-solving, cognitive flexibility, working memory, and ideomotor planning tests (40). Moreover, a large cohort study of the older adult population in the UK showed that LLD was associated with an 85, 65, and 152% increased risk of all-cause dementia, Alzheimer’s disease (AD), and vascular dementia, respectively, (41). Cumulative exposure to long-term depressive symptoms among older adults could contribute to predicting accelerated subsequent cognitive decline in a dose-response pattern (42).

Late-life depression is typically diagnosed according to the diagnostic criteria for adult depression. However, some clinical phenotypes of LLD and adult depression, including a unique central depressive symptom network, high rate of concomitant drug use, and co-existing cognitive impairments in LLD, differed. These phenotypes are associated with the pathogenesis of LLD.

## 4. The multiple pathogenesis of LLD

**FIGURE 2:**
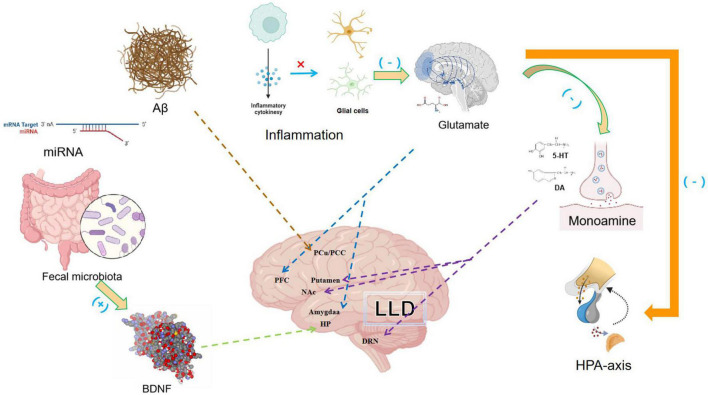
The multiple pathogenesis of late-life depression (LLD) include insufficient monoamine neurotransmission, increased inflammation, abnormal glutamate input, decreased neurotrophic factor production, the dysregulation of the hypothalamus-pituitary-adrenal (HPA) axis, age-related Aβ deposition and low diversity of the gut microbiome. The interplay between the different pathogenesis of LLD and the partial localization of some of the mechanisms in the brain was also shown. Pro-inflammatory cytokines can dysregulate the glutamate system, reduces the synthesis of serotonin and affect HPA axis.

The pathogenesis of LLD is complex. Multiple etiological hypotheses have been proposed to explain the underlying biological mechanisms of LLD (Figure 2).

The monoamine hypothesis of depression attributes the symptoms of major depressive disorders (MDDs) to imbalances in monoamine neurotransmitters including serotonin, dopamine, and norepinephrine (43). Low levels of serotonin transporters have been found in the frontal, temporal, and parietal cortical regions in patients with LLD and depressive symptoms (44). Moreover, the dysfunction of 5-HT_1A_ receptor activity in the brainstem region of the dorsal raphe nucleus has been observed in LLD (45). Dysfunction of the dopamine system causes alterations in the cognitive, positive valence, and sensorimotor systems, resulting in depressive symptoms. Low dopamine transporter binding potentials have also been found in the nucleus accumbens and putamen of patients with LLD compared to healthy controls. Thus, it is suggested that symptom of anhedonia found in LLD is associated with reduced dopamine neurotransmission in the reward system (46).

Furthermore, a positive correlation was observed between inflammation and LLD. Inflammatory cytokines may participate in the pathogenesis of LLD through multiple mechanisms. Inflammatory cytokines act directly on astrocytes and microglia, dysregulate the glutamate system, promote excitotoxicity, and activate indoleamine 2,3-dioxygenase, which reduces serotonin synthesis and increases kynurenine production. Increased kynurenine levels can result in oxidative stress, which consequently damages glial cells in the prefrontal cortex and amygdala. Moreover, inflammatory cytokines can affect the hypothalamus-pituitary-adrenal (HPA) axis, thereby destroying the inhibitory effects of glucocorticoids on inflammatory cytokines. Continuous activation of microglia can result in inefficient clearance of neurotoxic molecules, neuronal loss, and decreased neurogenesis (40, 47–49). A longitudinal study conducted over 2 years found that compared to non-depressive patients, the levels of IL-1β, IL-6, and IL-8 were higher in patients with LLD (50). IL-6 not only reduces the concentration of serotonin, but also damages the plasticity of synaptic nerves, which consequently results in cognitive impairment in LLD (51). Moreover, complaints and anhedonia, two important symptoms of depression, were positively correlated with C-reactive protein levels in patients with LLD, further supporting the link between inflammation and LLD (52).

Glutamate, a major excitatory neurotransmitter in the central nervous system, was found to have significantly higher glutamine-to-glutamate ratios at baseline in subjects with LLD. This ratio decreased in individuals with LLD over a 3 year follow-up, which correlated with a decrease in the severity of depression, suggesting that abnormalities in the glutamine–glutamate cycle may contribute to the pathophysiology of LLD (53). Glutamatergic synapses are excitatory synapses closely related to stress. Abnormal levels of N-methyl-D-aspartate (NMDA) receptors are also present in the brain of patients (54, 55). Ketamine, a non-competitive NMDA receptors agonist, has been used clinically to treat refractory depression. The safety and effectiveness of ketamine as an antidepressant for LLD have been proven in clinical trials, further supporting the involvement of NMDA receptors in the pathogenesis of LLD (56).

Neurotrophic factors are growth factors that regulate synaptic plasticity and neurotransmission, which can also play a role in LLD. Brain-derived neurotrophic factors (BDNF) can reverse defects in synaptic plasticity caused by stress, thereby enhancing flexibility against depression. BDNF levels in patients with LLD are significantly lower than those in healthy individuals (57, 58). And when compared to a control group, DNA methylation of BDNF in the LLD group was significantly higher (59). Higher BDNF methylation is independently associated with the prevalence and incidence of depression and major depressive symptoms (60). Longitudinal studies have reported 1−2% annual hippocampal atrophy among adults older than 55 years without dementia, and such age-related hippocampal deterioration and memory impairment may exacerbate depression in LLD patients (61).

The HPA axis is an important neuroendocrine system involved in controlling stress responses (62). Patients with LLD displayed significantly higher levels of basal cortisol during all phases of the diurnal cycle and higher levels of post-dexamethasone cortisol than healthy controls, suggesting that patients with LLD have HPA axis dysregulation (63). Moreover, patients with LLD have higher cortisol levels during morning awakening and a lower response to dynamic awakening, indicating that LLD responds to awakening stress. Older participants with depression showed a high degree of HPA axis activity dysregulation compared to younger adults, possibly because LLD lacks a buffering mechanism and displays higher HPA axis activity than its younger counterparts (63, 64).

The pathophysiological mechanisms of LLD and AD overlap. β-amyloid (Aβ) accumulation, a pathological hallmark of AD, has also been observed among patients with LLD. Aβ deposition results in a series of neurobiological processes that can damage neural networks related to depression (65). A community follow-up study of 270 older adults with un-impaired cognition showed that Aβ deposition was positively associated with an increase in anxiety-depressive symptoms (66). Moreover, older adults with depressive symptoms had more significant Aβ deposition in the anterior/posterior cingulate cortex than older adults without depressive symptoms (67). Furthermore, subjects with mild cognitive impairment and depression had severe Aβ deposits in the frontal cortex on both sides (68).

Age-related neurological conditions in LLD may also be a result of low-diversity gut microbiome (69). This is a common feature of biological aging, and age-dependent changes in the gut microbiota can manipulate neuroimmune responses (70). Gray matter volume (GMV) deficiency in marginal regions is strongly associated with LLD. And the great brain volume and increased gut diversity measures appear was positively associated with health, further informing the involvement of the brain-gut axis in LLD (71). In fact, rodents that receive fecal microbiome transplantation (FMT) from depressed humans can exhibit a heightened state of inflammation and increased anhedonia-like and anxiety-like behavior compared to those who receive FMT from healthy volunteers (72, 73). The baseline enrichment of Faecalibacterium, Agathobacter and Roseburia was positively associated with the treatment outcome of remission, indicating that the information from fecal microbiota might predict the response of antidepressants to LLD (74). Moreover, a randomized double-blind placebo-controlled multicenter trial found that probiotics containing Bifidobacterium bifidum BGN4 and Bifidobacterium longum BORI administered for 12 weeks significantly improved mental flexibility and alleviated stress among healthy older adults, supporting the regulation of LLD by affecting the gut microbiota (75).

The classic pathogenesis of LLD includes insufficient monoamine neurotransmission, increased inflammation, abnormal glutamate input, decreased neurotrophic factor production, and dysregulation of the HPA axis. Moreover, age-related Aβ deposition and low diversity of the gut microbiome might result in LLD with cognitive impairments. Therefore, further treatments should target LLD pathogenesis.

## 5. Pharmacological treatment of LLD

Selective serotonin reuptake inhibitors (SSRI) antidepressants are typically used to treat LLD. A study of 6,373 patients with LLD receiving SSRI antidepressants found that 50.7% of the patients achieved a reduction of at least 50% on the Hamilton depression scale (HAMD). This finding suggests that approximately 50% of older adults with MDD treated with SSRI antidepressants could exhibit symptom improvement when treated (76). According to the 2016 Canadian Network for Mood and Anxiety Treatments (CANMAT) guideline, first-line SSRI antidepressants recommendations for depression in older adults were duloxetine, mirtazapine, nortriptyline (level of evidence: level 1), bupropion, citalopram/escitalopram, desvenlafaxine, duloxetine, sertraline, venlafaxine, and vortioxetine (level of evidence: level 2) (54). Previously, there was a concern that paroxetine might cause adverse outcomes in the geriatric population owing to its anticholinergic properties. However, a recent study reported no increase in mortality, dementia risk, or cognitive measures in patients with paroxetine-treated LLD (77).

Although initial treatment, continuous treatment, and medication adjustment plans for treatment-resistant depression with LLD are similar to those for adult depression, several concerns should be considered when treating LLD with SSRI antidepressants (78). Aging affects pharmacokinetics, pharmacodynamics, and interactions between antidepressants. Moreover, liver function and kidney creatinine clearance rates continue to decline, and the pharmacodynamic sensitivity of older adults increase with age, resulting in reduced therapeutic effects, non-compliance to treatment, side effects, and poor tolerance of antidepressants in LLD (77). SSRI antidepressants can cause hyponatremia in older adults. Moreover, the incidence of these adverse effects can be greater than 10% when SSRI treatment is initiated for LLD (39). Additionally, the use of SSRI antidepressants was associated with a higher risk of hip fracture in older adults. In a large case-control study in Taiwan, the current use of SSRI antidepressants was associated with > two fold increase in the risk of hip fractures (79). Similarly, in a Danish nationwide population study, the prevalence of SSRI use among patients with hip fractures was approximately double that in the general population (80). In addition, patients with LLD and cognitive deficits do not respond well to antidepressants; the response of patients with LLD with executive function complaints to escitalopram was slower than that of patients with LLD without executive function complaints (81).

Antipsychotics (such as aripiprazole), mood stabilizers (such as lithium salt), and dopamine agonists (such as methylphenidate) are commonly used as synergists for adjuvant treatment of refractory LLD (82). Aripiprazole has been reported to improve the achievement of remission among LLD patients treated with venlafaxine, possibly *via* the partial activation of dopamine (83). Moreover, methylphenidate has been reported to potentiate the anti-depressive effects of citalopram, such as alleviation of depression severity, increased treatment response, and improved cognitive performance in patients with LLD (84). Additionally, 33.3% of patients with refractory LLD achieved remission using lithium salt synergistic therapy, and citalopram combined with lithium could be used to treat imipramine resistant LLD (85, 86).

Therefore, several novel antidepressant drugs have been developed for this purpose. The most studied novel antidepressants are the NMDA receptors antagonists, ketamine and memantine (40). Memantine has been used as an adjunct agent to citalopram in the treatment of LLD. The remission rate was higher in the escitalopram + memantine group than in the escitalopram-alone group. Moreover, a combination of memantine and escitalopram is well tolerated by patients with LLD (87). Ketamine is a racemic mixture of equal amounts of S-ketamine- and R-ketamine, which has a high affinity for the NMDA receptors esketamine (S-ketamine) and generates rapid and sustained antidepressant effects in patients with depression without notable side effects. A double-blind pilot study among older patients with treatment-resistant depression showed that ketamine was more effective than midazolam (88). Moreover, benzoate is a d-amino acid oxidase (DAAO) inhibitor and an indirect NMDAR enhancer. A recent randomized clinical trial found that benzoate decreases perceived stress, improves cognitive function, and enhances treatment adherence in patients with LLD (89).

Classical antidepressants are represented by SSRI antidepressants as the first-line treatment for LLD. However, these classical antidepressants are associated with various difficulties in LLD therapy, such as poor responsiveness to cognitive deficits, drug-drug interactions, and side effects among older adults. Although novel antidepressants with different mechanisms of action have been developed and are used as synergists for the adjuvant treatment of LLD, it is still necessary to investigate non-pharmacological treatments for this disorder.

## 6. Non-pharmacological treatment of LLD

Non-pharmacological treatments, which are safe and operable, have been widely used to treat LLD. Neuromodulation therapies use physical methods to implant treatment facilities in the body or outside the skin, to further adjust the function of the central nervous system and improve disease symptoms (90). The most common forms of neuro-modulatory therapy currently used for LLD include electroconvulsive therapy (ECT), transcranial magnetic stimulation (TMS), and light treatment (Table 1) (91–94).

**TABLE 1:** The most common neuromodulation therapy of LLD.

References	Intervention methods	Group	Efficacy results
(92)	ECT	Under 65 years old (*n* = 61); over 65 years old (*n* = 29)	ECT was more effective in older patients as compared to younger (*p* < 0.001). Most of the cognitive functions remained unchanged compared to baseline, whereas the outcomes of MMSE, Rey Auditory Verbal Learning Test (RAVLT) and Stroop tests showed greater improvements in the older compared to the younger group (all *p* < 0.05).
(93)	ECT	Self before and after control (1 week prior to ECT, weekly during ECT, after the sixth ECT course, and 1 week, 4 weeks, and 6 months after the last ECT course), *n* = 109.	MMSE scores improved significantly during the course of ECT and remained stable during the 6 months period after ending ECT for the total group. In the group of patients with a low MMSE score (< 24) at baseline, the MMSE score improved significantly during ECT, whereas in the group of patients with a normal MMSE score (≥ 24) at baseline, the score did not change significantly during ECT.
(91)	TMS	iTBS (self before and after control), *n* = 13.	Montgomery Asberg depression scale scores improved significantly from baseline to treatment-end. The flanker inhibitory control and attention test showed significant improvement in executive function from baseline to treatment-end.
(94)	Light therapy	Experimental group received light therapy (*n* = 34); control group received routine care without light therapy (*n* = 31)	The mean depression score in the experimental group decreased from 7.24 (SD 3.42) at pre-test to 5.91 (SD 3.40) at post-test, and had a significant reduction (*t* = 2.22, *P* = 0.03). However, there was no significant difference in depression score and sleep disruption between the experimental group and control group.

Electroconvulsive therapy stimulates the brain with a short and appropriate current, causing the patient’s brain cells to discharge synchronously, resulting in severe epileptic seizures. ECT is one of the most effective interventions for refractory depression. A randomized controlled trial on LLD, comparing the antidepressant effects of 6 weeks of ECT with those of 12 weeks of drug therapy found that patients who underwent ECT experienced faster symptom relief than the drug-treated patients (95). After the trial, the response rates in the two groups were 63.8 and 33.3%, respectively. Old age is also associated with a rapid response to ECT (96). ECT was more effective among older than younger patients. Moreover, no serious adverse events were observed in either group (92). Furthermore, most patients with LLD who underwent ECT had mild cognitive impairment within 1 week. However, 2 weeks after the end of ECT treatment, most cognitive functions remained unchanged compared to baseline functions (97). Mini-mental state examination (MMSE) scores tended to improve significantly 6 months after ECT in the event of baseline cognitive impairment with LLD (93). However, ECT requires an oxygen mask for spontaneous ventilation after anesthesia. During the COVID-19 pandemic, because of the possibility of virus transmission *via* bag-mask ventilation during the ECT procedure, many ECT units in hospitals worldwide were closed (Figure 1) (98).

Transcranial magnetic stimulation is an electrophysiological technique that uses a time-varying magnetic field to induce a time-varying electric field in the skull, resulting in enhanced brain metabolism and electrical nerve activity. TMS has proven to be effective for LLD treatment, with a response rate of approximately 20–50% (95). Moreover, a systematic review suggested that TMS is a safe and well-tolerated option for older patients with LLD with a relatively low percentage of side effects (12.4% in total) (99). Intermittent theta pulse stimulation (iTBS), a specialized form of TBS is effective in increasing cortical excitability and has been proven to be advantageous for patients with LLD and cognitive impairments (100). iTBS can reverse depression and executive function deficits in older patients (91). Moreover, a clinical case report suggested that iTBS can be used to treat LLD with comorbid mild cognitive impairments (101). Additionally, deep TMS can stimulate connections between subcortical structures and deep brain fibers. The remission rate of LLD patients in the deep TMS group was higher than in the sham TMS group, indicating the usefulness of deep TMS in the treatment of LLD (102).

Light therapies use sunlight at specific wavelengths. Light therapies can reduce symptoms of depression and sleep disruption in older adults residing in long-term care facilities. Bright light treatment can improve mood, enhance sleep efficiency, and increase the upslope melatonin level gradient of non-seasonal MDD among older adults with non-seasonal MDD (94, 103).

Psychotherapy has a moderate-to-strong effect on the improvement of depressive symptoms in LLD. Moreover, the therapeutic effects can last for at least 6 months (104). Cognitive behavioral therapy (CBT), problem-solving therapy (PST), interpersonal relationship therapy (IPT), and life review therapy are the main evidence-based psychotherapy methods for LLD treatment (40). In clinical practice, LLD patients in the context of hopelessness and age-related stressors have high suicide risk and great treatment challenges. Therefore, it is an urgent need to find alternative managements for treating such patients. The combination of psychotropic medications and the joy journal, a CBT-informed therapeutic intervention, could improve depressive symptoms of LLD associated with hopelessness during the COVID-19 pandemic (105).

Lifestyle behaviors may be an effective, low-cost intervention for improving the overall wellbeing of older adults. An active lifestyle, such as physical exercise or a balanced diet, could be a valid low-cost preventive strategy to counteract LLD (106). A study in South Korea suggested that it is important to encourage older individuals to exercise regularly to relieve depressive symptoms and that hand-grip strength may increase the effect of regular exercise on depressive symptoms among individuals 65 years and older (107).

Non-pharmacological treatments such as neuromodulation therapies and psychotherapy have been rapidly developed for the treatment of psychological disorders. ECT, TMS and phototherapy are among the most effective and safe non-pharmacological treatments for LLD. Importantly, neuromodulation therapy has proven to be advantageous for patients with LLD and cognitive impairments.

## 7. Complementary and integrative therapies

Many complementary and alternative medicine interventions, including Qigong, acupuncture, and traditional Chinese medicine were proved to be benefit for LLD patients, especially during the COVID-19 pandemic (108).

Qigong has gained worldwide popularity and was suitable for LLD patients who cannot go out during the COVID-19 pandemic. 10-week Tai Chi intervention decreased the depressive symptoms of participants aged 60−78 years during the COVID-19 pandemic (109). Moreover, there was significant improvement of Sahaj Samadhi meditation on the Hamilton rating scale for depression in old patients during the COVID-19 period (110). Traditional Chinese herb formulations, such as Chaihu Shugan San, Xiaoyao San, and Sini San were proved to be effective to reduce depressive symptoms in patients infected with COVID-19 virus (111). Moreover, acupuncture was proofed not only to relief primary and secondary depressive symptoms, but also to reduce the side effects of the medical treatments (112). And the safety and efficacy of acupuncture in the treatment of depression was also proved by a previous study (113).

Social distancing, quarantine, and limitations in outdoor activities have resulted in sarcopenia, which has been closely associated with LLD during the COVID-19 pandemic. Dietary essential amino acids (EAAs) could be a good alternative for counteracting against loss of muscle mass and function (114). Moreover, deficiencies in the EAAs isoleucine, leucine, and histidine could be used to predict depression in older women (115). The Mediterranean diet, characterized by an abundance of whole grains, plant foods, and olive oil, and a moderate intake of fish and wine, has been associated with a variety of disease outcomes. A longitudinal study of older residents in Chicago found that greater adherence to a Mediterranean diet was positively associated with a reduced number of newly occurring depressive symptoms (116).

## 8. Digit-based treatment of LLD

Following technological progress, modern digital health interventions have become mainstream in medical and healthcare services. During the COVID-19 pandemic, the conventional medical and healthcare service mode (hospital outpatient service and routine healthcare projects) has been “devastating,” whereas modern digital health intervention technologies have developed rapidly (Table 2 and Figure 1) (117–122).

**TABLE 2:** Digit-based treatment of LLD.

References	Intervention methods	Group	Efficacy results
(120)	Mobile app–based intervention	Self before and after control (*n* = 20).	30% (6/20) participants had improvements in depressive symptoms
(122)	iCBT	Home care workers-guided group (*n* = 11); the research assistant-guided groups (*n* = 5); the self-guided group (*n* = 5).	iCBT is a promising intervention for homebound older adults experiencing depression
(118)	Telephone-delivered CBT and telephone-delivered NST	CBT-T group, *n* = 70; NST-T group, *n* = 71.	Depressive symptoms (BDI) declined among all participants (CBT-T: −10.77; 95% CI, −12.73 to −8.81; NST-T: −7.54; 95% CI, −9.44 to −5.64). There was a significantly greater decline in depressive symptoms among participants in CBT-T (difference in improvement, −3.23; 95% CI, −5.97 to −0.50; *P* = 00.02).
(121)	iCBT	Treatment group (*n* = 29); delayed-treatment waitlist control group (*n* = 25).	Significantly lower scores on the PHQ-9 (Cohen’s *d* = 2.08; 95% CI: 1.38–2.72) were observed in the treatment group compared to the control group at post-treatment. The treatment group maintained these lower scores at the 3 months and 12 months follow-up time points.
(119)	MoodTech	One group received the online intervention without peer support, *n* = 11; another group received the online intervention with peer support, *n* = 23; final group waitlist control group, *n* = 11.	The peer support component appeared to benefit the participants by providing a medium for empathy, encouragement, appreciation, and affirmation, and collaborative problem-solving. Sharing of experiences on online support groups can lead to improved sentiment.

A socially assistive robot is an artificial intelligence system designed to interact with humans by following social behaviors and rules. Social robots are being used increasingly to provide personal support to older adults living in long-term care facilities. Moreover, they can help alleviate LLD when used in group activities (123).

An 8 weeks mobile app-based intervention with remote therapist support was found to decrease depressive and anxiety symptoms among community-dwelling middle-aged and older adults. Moreover, 45% of participants showed clinically significant improvements in either depressive or anxiety symptoms (120).

Internet-based cognitive behavioral therapy (iCBT) has also been used to treat patients with LLD (122). The potential efficacy and cost-effectiveness of iCBT in the treatment of LLD were examined in randomized interventions with telephone-delivered CBT or telephone-delivered non-directive supportive therapy (NST) for patients with LLD. After 4 months, both treatments resulted in a reduction in clinical symptoms. Moreover, telephone-delivered CBT yielded significantly greater improvements in depressive symptoms than NST (118, 121). An iCBT and mindfulness techniques were found to be effective in improving distress, depression and loneliness in older adults in Israel during the COVID-19 pandemic (124). MoodTech is a pilot study of internet-based interventions for LLD that provides a medium for empathy, encouragement, appreciation, affirmation, and collaborative problem-solving. Such internet-based interventions have proven to be viable and potentially cost-effective options for disseminating online treatments for LLD (119). Another study conducted a short-term intervention with internet-based cognitive-behavioral and mindfulness techniques for community-dwelling older adults in Israel during the COVID-19 pandemic. The study concluded that these interventions were effective in improving distress, depression, and loneliness in older adults and that different psychological techniques seemed to have different effects on these symptoms.

Digit-based treatments are rapidly being developed. In particular, following the devastating conventional medical supply during the COVID-19 pandemic, digit-based medical supply proved advantageous in providing personal services to patients with LLD and was proven to be effective and safe for LLD therapy.

## 9. Conclusion

This article reviews the research progress on LLD in epidemiology, clinical phenotype, pathogenesis, and treatment, focusing on the comparison of the differences between LLD and adult depression. Population aging, the COVID-19 pandemic, and societal stress significantly affect the emotional health of older adults, resulting in a high prevalence of LLD worldwide. The clinical phenotypes of LLD and adult depression differ. However, pathological mechanisms underlying LLD remain unclear. Traditional SSRI therapy yields poor response and side effects in the treatment of LLD. Although several forms of neuromodulation therapies, complementary and integrative therapies and digit-based treatments have been proven to be safe and effective in treating LLD, there is still a need to investigate the detailed pathogenesis of LLD and develop novel anti-LLD treatments.

## Author contributions

All authors listed have made a substantial, direct, and intellectual contribution to the work, and approved it for publication.
